# Study on Predictive Models for Differential Diagnosis of Diabetic Kidney Disease and Non-Diabetic Kidney Disease Based on Clinical and Biochemical Indicators

**DOI:** 10.7150/ijms.115709

**Published:** 2025-09-27

**Authors:** Pinning Feng, Xianlian Deng, Wenjia Gan, Yijiang Song, Yuyan Yang, Ruijie Zhang, Jin Li, Wenbin Lin

**Affiliations:** 1 Department of Clinical Laboratory, The First Affiliated Hospital of Sun Yat-Sen University, Guangzhou, Guangdong 510080, PR China.; 2 Mindray Medical International Limited, Shenzhen, Guangdong 518055, PR China.; 3 Department of Laboratory Medicine, Nanfang Hospital, Southern Medical University, Guangzhou, Guangdong 510515, PR China.; 4 Department of Nephrology, Shenzhen Third People's Hospital, No. 29 Bulan Road, Longgang District, Shenzhen, Guangdong Province, 518100, PR China.

**Keywords:** type 2 diabetes, diabetic kidney disease, non-diabetic kidney disease, differential diagnosis, predictive model

## Abstract

**Background:** The differential diagnosis of diabetic kidney disease (DKD) from non-diabetic kidney disease (NDKD) presents significant challenges in clinical practice, as current diagnostic methods, such as renal biopsy, are invasive and lack specificity. This study aims to develop a non-invasive predictive model based on clinical and biochemical indicators to enhance diagnostic accuracy in distinguishing DKD from NDKD. The model is designed to serve as a decision-support tool for clinicians, improving renal health management in patients with type 2 diabetes mellitus (T2DM)**.**

**Methods:** A retrospective examination of data was executed. Clinical characteristics and laboratory data of T2DM patients who underwent renal biopsy at The First Affiliated Hospital of Sun Yat-sen University, spanning January 2015 to September 2023, were collated and stratified into a training cohort (January 2020 to September 2023) and an internal validation cohort (January 2015 to December 2019). A distinct analysis was conducted for patients with renal transplants within the validation cohort. Partial case data from Shenzhen Third Hospital (January 2022 to July 2025) and Southern Hospital (January 2018 to December 2023) were used as external validation cohort. The training cohort data underwent both univariate and multivariate regression analyses to formulate a predictive probability model, which was subsequently subjected to validation against the validation cohort. The efficacy of the model was meticulously assessed through metrics such as the area under the ROC curve, calibration plots, DAC, and the Hosmer-Lemeshow goodness-of-fit test.

**Results:** The study encompassed a total of 1091 T2DM patients, including 385 with DKD, 585 with NDKD, and 121 with a concomitant diagnosis of DKD and NDKD, denoted as MIX. Membranous nephropathy was identified as the predominant pathological entity in both NDKD and MIX cases. The probability model incorporated six variables: gender, age, diabetes duration, diabetic retinopathy status, serum uric acid, and low-density lipoprotein levels. The model demonstrated robust discrimination and calibration capabilities for patients without renal transplants but exhibited diminished applicability for those with renal transplants.

**Conclusion:** The research successfully established a model capable of accurately forecasting the likelihood of NDKD in the renal biopsy findings of T2DM patients. However, the model's applicability to patients with renal transplants is constrained, suggesting that future research endeavors should focus on enhancing the model to encompass a more diverse patient demographic.

## Introduction

Diabetes mellitus (DM) is a chronic metabolic disease that persistently affects hundreds of millions of people worldwide, with its incidence and prevalence steadily rising. It is projected that by 2045, the number of adult DM patients globally will increase from the current approximately 537 million to 783 million, with the majority being type 2 diabetes mellitus (T2DM) patients^1^-^3^. This growth trend not only represents a significant public health challenge but also indicates that the burden of related complications will further intensify. Diabetic kidney disease (DKD) is one of the most common complications of DM and a crucial factor leading to chronic kidney damage and end-stage renal disease^4^, ^5^. However, in clinical practice, it is difficult to distinguish between DKD, non-diabetic kidney disease (NDKD), and a mixed type (MIX) of DKD and NDKD^6^, ^7^. Studies have found that compared to DKD patients, NDKD or MIX patients have better renal prognosis and survival rates after timely and effective treatment. If misdiagnosed as DKD, it may lead to missed opportunities for disease-specific treatments, such as immunosuppressants or biologics that improve renal prognosis^8^. Therefore, distinguishing DKD from NDKD is crucial for the treatment planning and prognosis of DM patients with renal impairment.

Currently, non-invasive and specific diagnostic markers for DKD have not been discovered, making renal biopsy the gold standard for diagnosing DKD^9^. However, in the early stages of the disease, pathological features are not yet typical. There are diagnostic differences among doctors at different levels of hospitals, and different diagnoses may occur within the same tissue, leading to potential misdiagnosis or missed diagnosis. Moreover, renal biopsy is an invasive examination that can result in a series of complications such as perirenal hematoma post-operation^10^. It also has contraindications and is not suitable for every patient. Some hospitals with inadequate conditions do not have the capability to perform this examination. To date, there are no formal practice guidelines regarding the timing of renal biopsy in DM patients, indicating the necessity to develop a new tool to differentiate DKD from NDKD.

Although international guidelines such as the KDOQI guidelines provide recommendations for distinguishing DKD from NDKD, these guidelines have low specificity in China, at only 40.63%^11^. Several parameters currently used in clinical practice, such as the diabetes duration and levels of glycated hemoglobin, have inconsistent predictive values in different studies, and the predictive accuracy of single indicators is limited, whereas models include many factors^12^-^16^. Recent advances in metabolomics research have increasingly demonstrated that serum and urinary metabolic markers play crucial roles in the prognosis, diagnosis, and treatment monitoring of various kidney diseases. These biomarkers may also possess potential value in differentiating diabetic kidney disease (DKD) from non-diabetic kidney disease (NDKD). However, translation into clinical practice would require further comprehensive validation and evidence-based verification^17^-^20^. If a simple predictive tool can be developed based on clinical and common biochemical indicators to preliminarily predict the probability of having NDKD at an early stage or initial patient visit, it would assist doctors in more accurately distinguishing between DKD and NDKD early on.

This study aims to develop and validate a non-invasive differential diagnosis predictive scoring model for the differential diagnosis of DKD and NDKD based on clinical and biochemical indicators. The development and application of this model will provide a convenient decision support tool for clinical practice, potentially improving kidney health management in DM patients. However, the applicability of the model to kidney transplant patients is limited, indicating that one future research direction may be to further optimize the model to cover a broader patient population.

## Methods

### Study Subjects

Clinical, laboratory examination, and pathological data of a total of 1091 T2DM patients who underwent renal biopsy were collected. At The First Affiliated Hospital of Sun Yat-sen University from January 2015 to September 2023, data from January 2020 to September 2023 (n=330) were used as the training set, and data from January 2015 to December 2019 (n=360) were used as the internal validation set. Considering the special nature of kidney transplant patients, they were not included in the training set. However, to evaluate the model's applicability to kidney transplant patients, they were separately analyzed in the validation set. The different validation sets were determined based on whether the patients had undergone kidney transplantation: non-kidney transplant group (n=287), kidney transplant group (n=73), and combined non-kidney transplant and kidney transplant group (n=360). Data from Shenzhen Third Hospital (January 2022 to July 2025, n=201) and Southern Hospital (January 2018 to December 2023, n=200) were used as the external validation set1 and set2 respectively. The included patients had to meet the basic inclusion criteria, namely, being over 18 years old at the time of renal biopsy and having been clinically diagnosed with T2DM. Patients with incomplete or unclear medical records or history, missing retinal examination results, and those with malignant tumors, severe infections, or systemic diseases such as allergic asthma and AIDS were excluded. This rigorous screening mechanism ensured the quality of the samples and the accuracy of the study. The pathological examination and diagnosis were conducted by experienced experts from the institution, using light microscopy, immunofluorescence, and electron microscopy results of the specimens. Patients were classified into three groups: pure DKD, MIX (i.e., DKD coexisting with other non-diabetic kidney diseases), and pure NDKD. MIX patients were excluded from the training set to ensure the accurate establishment of the model^21^.

### Data Collection

The construction of the model considered various factors, including demographic baseline data such as sex, age, height, weight, body mass index (BMI); clinical history such as diabetes duration, diabetic retinopathy (DR); blood biochemistry such as serum creatinine level (Scr), blood urea concentration (UREA), blood uric acid content (UA), estimated glomerular filtration rate (eGFR) based on Scr, serum albumin level (ALB), immunoglobulin A concentration (IgA), four lipid profile components (cholesterol/triglyceride/high density lipoprotein cholesterol/low density lipoprotein cholesterol, TC/TG/HDL-C/LDL-C), fasting blood glucose concentration (Glu), glycated hemoglobin level (HbA1c); urine biochemistry such as urinary κ-chain (U_κ), urinary immunoglobulin G (U_IgG), 24-hour urinary total protein amount (24hUTP); routine blood tests such as red blood cell count (RBC), hemoglobin concentration level (HGB), and hematocrit (HCT). These factors were collected during the hospital admission when the renal biopsy was performed, and the data were obtained through electronic records or telephone follow-ups. A total of 10 variables with a missingness rate exceeding 30% were excluded from statistical analysis. These included serum variables: Cystatin C (CysC), Retinol-Binding Protein (RBP), β2-Microglobulin (β2MG), Complement C1q, C1q Antibody, Homocysteine (HCY); and urinary variables: Urinary Creatinine (UCREA), Urinary Albumin-to-Creatinine Ratio (UACR), Urinary N-Acetyl-β-D-Glucosaminidase (UNAG), Urinary Retinol-Binding Protein (URBP). For the 8 variables with a missingness rate below 30% - including serum variables: UA, HbA1c, TC, TG, HDL, LDL; and urinary variables: U_κ, U_IgG- mean imputation was employed to ensure data completeness and analytical accuracy.

### Indicator Definitions

The diagnosis of T2DM followed the authoritative standards issued by the American Diabetes Association^22^. The term “diabetes duration” specifically refers to the period from the patient's initial diagnosis of T2DM to the time of hospital admission for renal biopsy. Hypertension was defined as having a systolic blood pressure exceeding 140 mmHg or a diastolic blood pressure above 90 mmHg without the use of any antihypertensive medications^23^. The diagnosis of DKD and NDKD was based on microscopic renal tissue manifestations, including but not limited to glomerular enlargement, excessive mesangial matrix proliferation, mesangial lysis, and the formation of characteristic Kimmelstiel-Wilson nodules, microaneurysm-like expansions, exudative lesions, glomerular vascular pole neovascularization, and tubulointerstitial lesions^24^, ^25^.

### Statistical Methods

Statistical analysis was conducted using SPSS version 27.0 and R version 4.3.1. A *P*-value of less than 0.05 was considered to indicate a statistically significant difference. Normally distributed data were presented as mean ± standard deviation (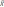
±s) and compared between groups using one-way analysis of variance (ANOVA). Non-normally distributed data were expressed as median (Q1, Q3) and the Mann-Whitney U test was used to compare differences between groups. Categorical count data were described using frequencies (percentages) and the chi-square (χ2) test was used to compare differences between groups. To assess the association between variables and the progression of T2DM kidney injury to NDKD, a logistic regression model was used to estimate the odds ratios (ORs) of these variables. Variables with a *P*-value less than 0.05 in the univariate analysis were included in the multivariate analysis. The selection of predictor variables was based not only on statistical results but also on existing research findings and clinical insights. Variables were further refined using bidirectional stepwise method to identify the model with minimal Akaike Information Criterion (AIC). The model was established using the training set and further validated using the validation set. To comprehensively evaluate the model's discrimination, accuracy, and reliability, methods such as C-statistic and calibration curves were used. Additionally, the Hosmer-Lemeshow test was applied to assess the calibration of the model, i.e., the agreement between predicted values and actual observations.

## Results

### Characteristics of Pathological and Clinical Baseline Data

#### Pathological Results of Patients in the Training and Validation Sets

The study covered a total of 1091 T2DM patients with kidney damage who underwent renal biopsy. Analysis of renal biopsy data showed that among the 330 patients in the training set, approximately 39.1% (129 cases) had pure DKD, about 48.8% (161 cases) had NDKD, and 12.1% (40 cases) had MIX. Among the patients with pure NDKD, membranous nephropathy was the most common pathological type (about 34.8%), followed by IgA nephropathy (18%) and hypertensive nephropathy (8.7%). For MIX patients, hypertensive nephropathy (35%) was the most common, followed by membranous nephropathy (32.5%) and IgA nephropathy (12.5%). The corresponding pathological types and distributions in the training and validation sets are detailed in Supplementary Table 1.

#### Clinical Baseline Characteristics of Patients in the Training and Validation Sets

Only the data from the DKD and NDKD groups were used to avoid the impact of confounding factors^21^. Comparing the DKD and NDKD groups in the training set, the following indicators showed statistical differences: In the DKD group, the proportion of males, height, diabetes duration, proportion of DR, SBP (Systolic Blood Pressure), PP (Pulse Pressure), Scr, UREA, UA, ALB, Glu, HbA1c, and U_κ were higher; age, eGFR, TC, LDL-C, 24hUTP, RBC, HGB, and HCT levels were lower. There were no statistical differences between the two groups in BMI, DBP (Diastolic Blood Pressure), IgA, TG, HDL-C and U_IgG. Detailed clinical baseline characteristics of the training and validation sets are shown in Table 1A,B,C.

### Model Development and Validation

Through univariate regression analysis, 24 important variables were identified, including demographic and clinical baseline factors such as sex, age, height, diabetes duration, SBP, PP, DR; renal function markers in blood such as UA, UREA, CREA, eGFR; blood lipids such as TC, HDL, LDL; blood glucose monitoring markers such as Glu, HbA1c; urine biochemistry markers such as U-κ light chain, 24-hour mALB; and routine blood tests such as RBC, HGB, HCT. Subsequently, multivariate regression analysis identified six statistically significant variables: sex, age, diabetes duration, UA, DR and LDL. Using a forward and backward stepwise method (AIC=221.67), incorporating variables with *P*<0.05 and those empirically relevant to outcomes in clinical practice or based on published literature, such as DR, BMI, the final prediction model variables were selected based on simplicity and objectivity without significantly affecting model efficiency. The final variables included in the predictive model were: age, sex, diabetes duration, LDL, UA, and DR. The results of univariate and multivariate logistic regression analyses are detailed in Table 2. After adjusting for other variables in the model, males had 12.577 times the odds of developing NDKD compared to females (OR: 12.577; 95% CI: [1.144, 169.707]). For each one-year increase in age, the odds of NDKD increased by 2.1% (OR: 1.021; 95% CI: [1.000, 1.119]). Each additional month of diabetes duration was associated with a 1.2% reduction in the odds of NDKD (OR: 0.988; 95% CI: [0.976, 0.999]). Per unit increase in UA level, the odds of NDND increased by 123.2% (OR: 2.232; 95% CI: [1.096, 5.241]). Patients with DR had 0.014 times the odds of developing NDKD compared to those without DR (OR: 0.014; 95% CI: [0.000, 1.000]). Each unit increase in LDL was associated with a 51.6% reduction in the odds of NDKD (OR: 0.484; 95% CI: [0.275, 0.749]).

The specific formula for the predictive model is P=(exp(A))/(1+exp(A))​, where P is the probability of NDKD in T2DM patients based on renal biopsy pathology results, and A is calculated as follows:

A=-2.075984+1.678152×sex+0.029880×age-0.16316× diabetes duration -0.001747×UA+0.155641×LDL+2.324066×diabetic retinopathy

In this formula, for continuous variables, the actual test results are directly substituted into the equation, and diabetic retinopathy detection results are treated as binary variables, with 1 indicating the presence of diabetic retinopathy and 0 indicating its absence.

**Figure 1:**
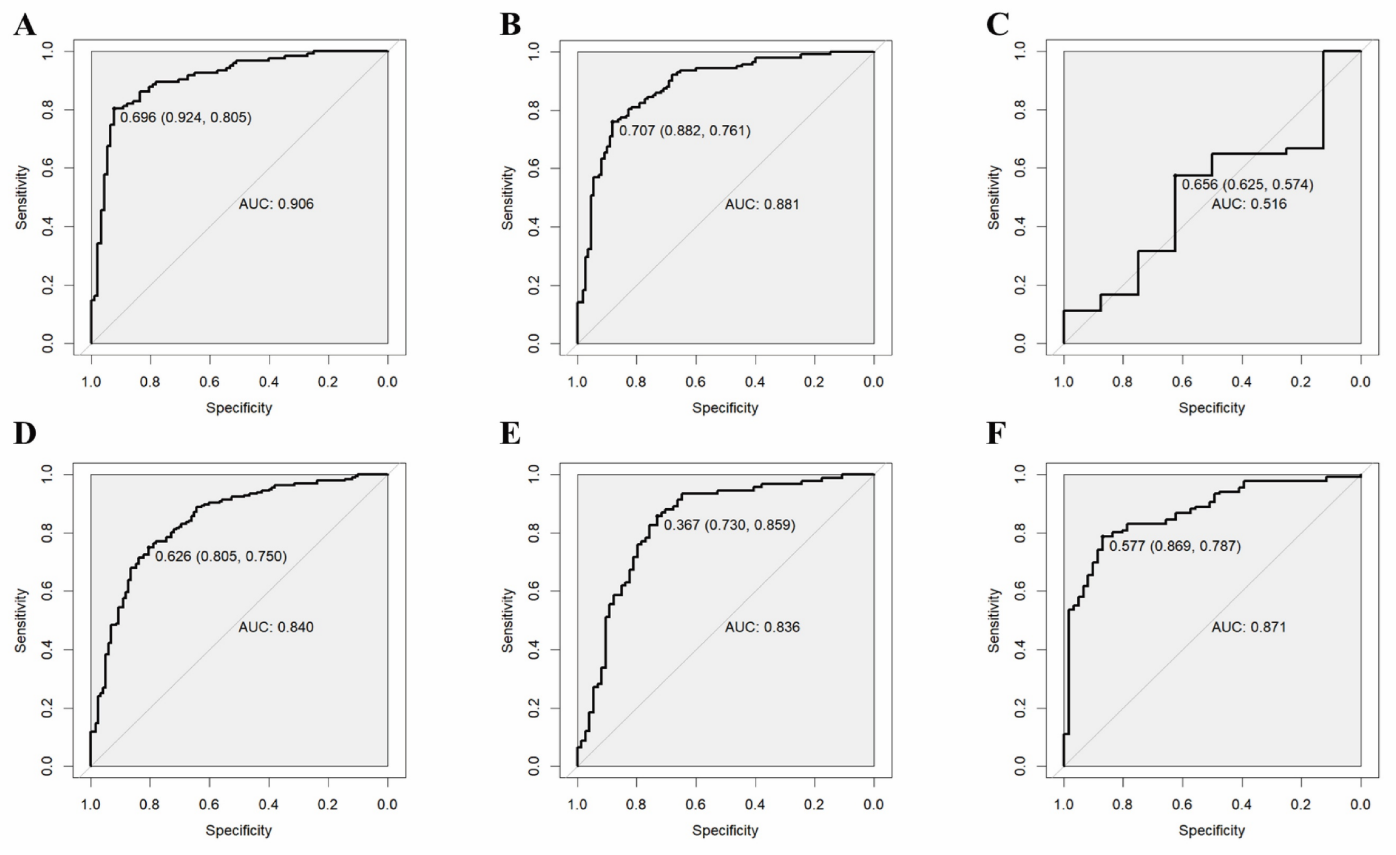
ROC curves of the predictive model in the training set (A) and internal verification sets of non-renal transplant group (B), renal transplant group (C), non-renal transplant + renal transplant group (D), external sets of Shenzhen Third People's Hospital (E) and Nanfang Hospital of Southern Medical University (F).

**Figure 2:**
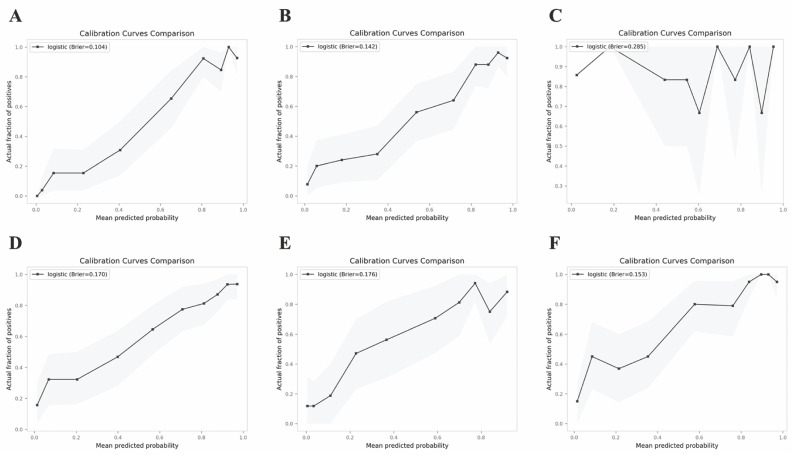
Calibration curves of the predictive model in the training set (A) and verification sets of non-renal transplant group (B), renal transplant group (C), non-renal transplant + renal transplant group (D), external sets of Shenzhen Third People's Hospital (E) and Nanfang Hospital of Southern Medical University (F).

**Figure 3:**
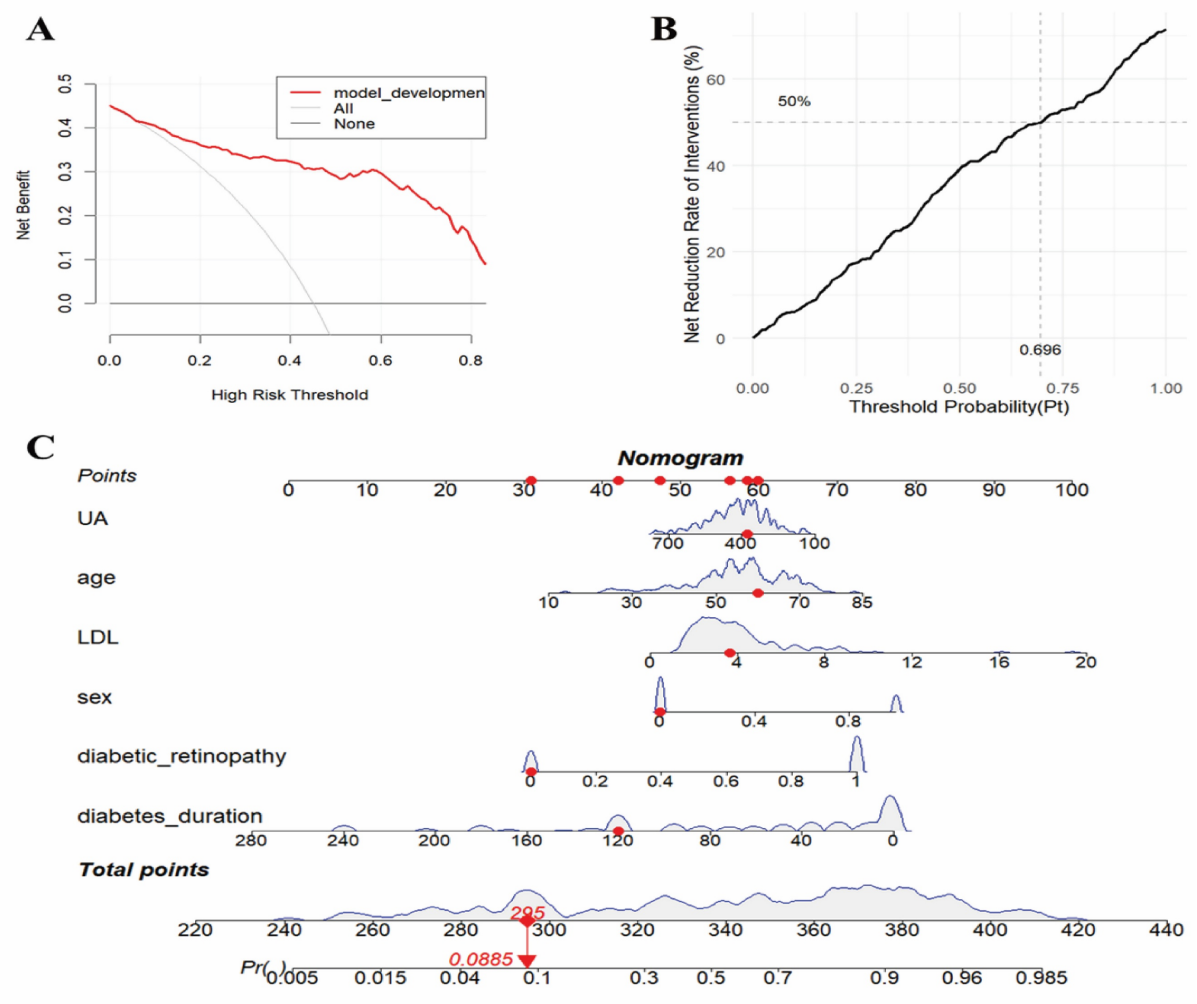
Clinical decision curve analysis (a-b) and prediction probability nomogram (C) of NDKD in T2DM patients undergoing renal biopsy.

The validation results on the verification set indicate that the established predictive probability model demonstrates good discrimination and calibration in both the training dataset and the non-renal transplant group verification dataset. For the training dataset, the C-statistic (AUC) is 0.906 (95% CI 0.8643-0.9478), with a sensitivity of 80.5%, specificity of 92.4%, and a cutoff value of 0.696. The positive likelihood ratio (PPV) is 93.4%, and the negative likelihood ratio (NPV) is 80.0%. The AUC for the non-renal transplant verification dataset is 0.881 (95% CI 0.8379-0.9342), with a sensitivity of 76.1% and specificity of 88.2%. However, the model performs poorly in the renal transplant dataset, with an AUC of only 0.516 (95% CI 0.2934-0.739) and a sensitivity of 57.4% and specificity of 62.5%. The model's performance declines in the combined non-renal transplant and renal transplant verification dataset, with an AUC of 0.84 (95% CI 0.7944-0.8862), sensitivity of 75.0%, and specificity of 80.5%. The AUC for the external validation set 1 is 0.836(95% CI 0.7704-0.9008), with a sensitivity of 85.9% and specificity of 73.0%, and the external validation set 2 is 0.871(95% CI 0.8190-0.9221), with a sensitivity of 78.7% and specificity of 86.9%, which had good performance. The Hosmer-Lemeshow test results show no statistical difference between predicted and observed probabilities in the training dataset (*P*=0.287), non-renal transplant verification dataset (*P*=0.126), external validation set 1(*P*=0.895), external validation set 2(*P*=0.268), but there is a statistical difference in the renal transplant verification dataset (*P*=7.81E^-11^), the combined non-renal transplant and renal transplant verification dataset (*P*=0.003). The performance of the predictive model across the training, internal validation, and external validation sets is detailed in Table 3, and ROC curves are detailed in Figure 1, while calibration curves are detailed in Figure 2. Results of the clinical utility evaluation DCA showed that when the threshold probability ranged between 0.05-0.80, patients achieved relatively high clinical net benefit. At a threshold probability of 0.696, approximately 20% of patients obtained net benefit (Figure 3 A). By applying this model for early diagnosis of NDKD, the renal biopsy rate could potentially be reduced by 50.0% (Figure 3 B).

### Clinical Decision Curve Analysis and Drawing and Application of Nomograms

The clinical utility of the model was validated and evaluated using Decision Curve Analysis (DCA) (see Figure 3 A-B). A probability model for predicting NDKD in T2DM patients has been successfully constructed. Using R language version 4.3.1, scientific and reasonable values were assigned to each risk factor within the model, and corresponding nomograms were drawn (see Figure 3 C). These nomograms can be transformed into applications such as apps, WeChat mini-programs, and web calculators to provide recommendations to healthcare workers and patients in a more convenient form. This tool can be used in three steps: first, assign scores to each variable based on the patient's results corresponding to the first row; then, add up the scores of all variables to obtain the total score; finally, by matching the total score to the probability scale at the bottom of the nomogram, healthcare workers or patients can determine the risk probability of developing NDKD.

## Discussion

This study included 1091 patients with T2DM, with pure DKD patients accounting for 35.3%, pure NDKD patients nearly half at approximately 53.6%, and MIX patients comprising 11.1%. In patients with NDKD and MIX, common pathological types include membranous nephropathy, IgA nephropathy, hypertension-induced renal injury, minimal change disease, and focal segmental glomerulosclerosis, with proportions and type distributions comparable to other studies in Asia^11^, ^15^, ^21^, ^26^-^30^. This indicates the representativeness of our study population and biopsy indications, laying the foundation for the extrapolation of the model. Different pathological diagnoses can affect treatment goals and regimens, such as individualized treatments with immunosuppressive agents or antiviral drugs, which may alter the progression of renal disease, underscoring the importance of identifying potential NDKD.

This study aimed to establish and validate a predictive model based on clinical and objective hematological indicators to differentiate between DKD and NDKD in patients with T2DM. The results of the study are partially consistent with previous relevant research. Zhou *et al.*^21^ initially established a model consisting of diabetes duration, systolic blood pressure (SBP), HbA1c, haematuria and diabetic retinopathy (DR) as the predictors. Liu *et al.*^30^ updated the model by adding HGB, finding that shorter diabetes duration, absence of DR, lower HbA1c, lower SBP, higher HGB, and the presence of haematuria are relatively good predictors for differentiating between DKD and NDKD, which is consistent with the meta-analysis results of Marco *et al.*^7^. In recent years, studies by Liu *et al.*^11^, ^26^, Li *et al.*^27^, Yang *et al.*^28^, Jiang *et al.*^29^, Chen *et al.*^15^ and María José Soler *et al.*^16^ have shown that younger age, longer diabetes duration, presence of DR, absence of haematuria, presence of anemia, lower HGB, UA, ALB, eGFR, proteinuria levels, and higher fasting Glu, HbA1c, CysC, and DBP levels are important independent predictors for the occurrence and progression of DKD, serving as important scoring indicators for differentiating between DKD and NDKD. In this study, sex, age, diabetes duration, DR, LDL, and UA all showed high predictive value in both univariate and multivariate regression analyses.

Considering the specificity of kidney transplant patients, renal transplant patients were not included in the training set, but to assess the applicability of the model in renal transplant patients, renal transplant patients were included in the verification set for separate analysis. The results showed that the predictive model established in this study has low clinical efficacy in distinguishing between DKD and NDKD in renal transplant patients, so it is not recommended to use this model in renal transplant patients. Possible reasons for this may include: (1) The diabetes duration in clinical settings is the time from the first diagnosis of DM to the time of renal biopsy due to kidney damage, but in the transplant group, this indicator may have errors due to the transplantation, and renal function results cannot be relatively sustained. (2) Renal transplant patients have issues with autoimmune rejection and ischemic damage to kidney vessels after transplantation. (3) Renal transplant patients use immunosuppressive drugs after surgery, which may cause damage to kidney function and may also lead to secondary cardiovascular diseases, resulting in deviations in the measurement of some biochemical results. However, in future studies, to extend the model's applicability to the transplant population, we will develop a dedicated discriminative diagnostic model for diabetic patients with renal insufficiency who have undergone kidney transplantation. This model will incorporate key transplant-specific predictors, such as immunosuppressive therapy status, graft vintage, donor type, and history of rejection. It will be trained and validated within large multi-center transplant cohorts to ensure robustness and generalizability. In this study, it was found that the model's efficacy reached 0.84 in the verification set of the non-renal transplant + renal transplant group, compared to 0.881 in the non-renal transplant group, and 0.516 in the renal transplant group verification set, indicating that this model is not applicable for predicting DKD and NDKD in renal transplant patients. Moreover, the AUC values of the external validation cohorts were 0.836 and 0.871, indicating that the model was also applicable in external hospitals.

This study has several strengths in building the model. Firstly, the discriminative diagnostic predictive model established in this study includes relatively fewer indicators and can effectively differentiate between DKD and NDKD in patients with T2DM in the non-renal transplant group, with a validated efficacy of up to 0.881. Secondly, a significant advantage of this study is the inclusion of a relatively larger sample size and two external validation cohorts, which not only enhances the universality of the research results but also improves the credibility of the validation results. Finally, the predictive model established in this study is based on objective biochemical indicators, incorporating factors such as age, sex, diabetes duration, and DR, which can be easily obtained through simple blood tests. The results are presented in the form of a nomogram, which are intuitive and convenient, greatly facilitating clinical application and avoiding the need for complex regression equations as proposed in previous studies^21^, ^28^, ^30^. Through this effective predictive tool, doctors can provide more personalized treatment plans for patients aimed at improving kidney prognosis, thereby optimizing treatment outcomes and quality of life for patients.

This tool is poised to significantly alter clinical patient management pathways. Specifically, in primary care settings lacking renal biopsy capabilities, when encountering diabetic patients with renal impairment, the tool enables prediction of NDKD probability. This allows physicians to proactively advise patients to seek timely renal biopsy at tertiary hospitals for definitive diagnosis and etiology-specific treatment, thereby effectively delaying disease progression and improving prognosis. In nephrology departments of tertiary hospitals equipped with biopsy facilities, for diabetic renal impairment patients with contraindications to biopsy (e.g., coagulation disorders), the tool provides predictive NDKD probability assessments. This serves dual purposes: first, it establishes an evidence-based foundation for empirical treatment, enhancing clinicians' decision-making confidence and facilitating early intervention to retard disease progression and improve outcomes; second, it reduces unnecessary biopsy procedures, thereby optimizing resource allocation and alleviating the economic burden on both patients and the healthcare system.

This study offers significant insights into differential diagnosis models, yet several methodological aspects warrant further consideration. The use of retrospective clinical data, while efficient for analyzing real-world parameters, underscores the importance of prospective validation to confirm these findings. Our multi-center approach ensured consistent data collection, but expanding to more multicenter collaborations could enhance the model's applicability across diverse healthcare environments. While our focus on distinguishing NDKD patterns reflects current diagnostic priorities, broadening sample cohorts may allow future exploration of overlapping renal conditions. Although established biomarkers were included in feature selection, emerging tubular injury markers should be investigated in subsequent studies. The multifactorial complexity of diabetic renal injury also suggests potential benefits of integrating genomic and lifestyle factors into advanced predictive frameworks.

## Conclusions

In conclusion, this study successfully established and validated a simple probability assessment model to predict NDKD based on renal biopsy pathology results for patients with T2DM. The model's accuracy is acceptable in patients without renal transplantation but is not applicable to renal transplant patients. If future large-scale multicenter validation studies can be conducted, the model may be widely promoted and used. To assist clinical decision-making, corresponding applications, webpages, or apps can be developed to provide a convenient and non-invasive tool for the differential diagnosis of DKD and NDKD, helping healthcare workers balance the risks and potential benefits faced by T2DM patients undergoing renal biopsy.

## Supplementary Material

Supplementary tables.

## Figures and Tables

**Table 1A:** Clinical Baseline Characteristics of Patients in the Training and Validation Sets

Clinical indicator	Training set					Validation set (Non-Transplant Group)				
	Total (n=290)	DKD group (n=129)	NDKD group (n=161)	Statistic (χ2/F/Z value)	*P* value	Total (n=252)	DKD group (n=110)	NDKD group (n=142)	Statistic (χ2/F/Z value)	*P* value
Male [cases (%)]	202(69.7)	105(81.4)	97(60.2)	0.006^a^	0.001	175(69.4)	90(81.8)	85(59.9)	0.313a	0.001
Age (years)	54(21,83)	53(23,77)	55(21,83)	-0.674	0.049	49(25,77)	51(30,70)	48(25,77)	-0.961	0.036
Height (m)	1.65(1.4,1.83)	1.67(1.4,1.81)	1.64(1.45,1.83)	0.005	0.001	1.65(1.44,1.86)	1.67(1.4,1.86)	1.63(1.44,1.81)	0.006	0.001
Weight (kg)	67.7(40,108)	68.3(40,9.78)	67.1(41,108)	-1.022	0.307	65.85(38.9,146)	68.26(42.0,114.2)	63.98(38.9,146)	-0.500	0.417
BMI (kg/m²)	24.82(17.09,36.51)	24.56(17.67,32.87)	25.02(17.09,36.51)	-0.396	0.570	24.08(20.87,29.37)	24.47(21.63,28.20)	23.78(21.46,27.63)	-0.970	0.315
Diabetes time(months)	73(0.1,336)	109(0.3,336)	44(0.1,240)	-4.674	0.001	62(1, 312)	98(1, 312)	33(1, 240)	-3.085	0.002
SBP (mmHg)	143±26	146±26	139±24	1.491	0.019	141±23	145±24	138±22	1.567	0.071
DBP (mmHg)	84±11	82±11	85±12	-0.768	0.245	86±14	86±12	86±15	-0.019	0.883
PP (mmHg)	59(17,108)	64(18,108)	55(17,100)	-2.842	0.001	57(35,65)	62(40,69)	53(33,59)	-2.472	0.005
UA (μmol/L)	418±114	442±104	399±119	-0.001	0.002	421±118	434±106	411±126	-0.790	0.376
UREA (mmol/L)	13.38(2.2,74.6)	16.15(4.6,74.6)	11.17(2.2,35.1)	-2.750	0.001	14.47(5.91,19.32)	19.27(6.50,19.80)	10.75(5.24,18.60)	-1.780	0.025
Scr (umol/L)	270(37,1303)	341(43,1120)	214(37,1303)	-2.392	0.001	255(96,445)	315(97,346)	208(90,245)	-0.910	0.363
DR	105(36.2)	85(65.9)	20(12.4)	-3.712	0.000	74(29.4)	67(60.9)	7(4.9)	-0.130	0.001
eGFR (mL/min/1.73 m^2^)	47.2(4.58.161.34)	35.3(6.2,152.16)	56.73(4.58,161.34)	-2.381	0.012	54.59(48.82,81.47)	40.92(46.37,72.90)	65.18(48.82,85.83)	-0.660	0.284
IgA (g/L)	2.59(0.27,8.72)	2.60(0.44,5.99)	2.57(0.27,8.72)	-2.175	0.238	2.47(2.15,3.31)	2.52(2.07,3.13)	2.42(2.27,3.50)	-1.380	0.104
ALB (g/L)	30.8±7.5	32.0±5.7	29.7±8.5	-1.780	0.010	31.8±7.8	33.1±6.7	30.8±8.4	-1.959	0.003
TC (mmol/L)	6.21(2.1,28.7)	5.58(2.5,20.6)	6.7(2.1,28.7)	-0.873	0.004	6.10(4.11,5.65)	5.64(4.08,5.81)	6.46(4.18,6.92)	-1.020	0.163
TG (mmol/L)	2.52(1.25,3.15)	2.31(1.25,3.14)	2.68(1.21,3.20)	-1.076	0.177	2.18(1.28,2.34)	2.00(1.10,2.20)	2.31(1.85,2.66)	-2.010	0.022
LDL-C (mmol/L)	1.25(0.86,1.29)	1.15(0.93,1.20)	1.32(0.84,1.33)	-0.095	0.008	1.74(0.86,1.94)	2.18(0.93,2.34)	1.41(0.84,1.54)	-2.381	0.017
HDL-C (mmol/L)	3.87(2.15,3.94)	3.44(2.12,3.70)	4.21(2.16,4.24)	-1.288	0.277	3.74(2.31,3.89)	3.37(2.15,3.81)	4.02(2.50,4.14)	-0.020	0.009
Fasting Blood Glu (mmol/L)	7.10(5.64,8.49)	7.49(6.12,9.11)	6.79 (5.53,7.40)	-3.938	0.002	6.54(5.52,9.79)	7.10(5.38,10.72)	6.10(5.78,8.15)	-0.040	0.042
HbA1c (%)	6.99(6.2,8.7)	7.21(6.5,8.8)	6.82(6.1,7.4)	-3.907	0.034	6.54(6.6,8.8)	7.10(7.30,9.40)	6.72(6.55,8.40)	-1.002	0.045
U_κ (mg/L)	7.87(2.08,20.8)	8.74(2.73,20.13)	7.17(2.08,20.8)	-3.653	0.000	8.45(1.28,64.84)	8.98(3.15,27.01)	8.05(1.28,64.64)	-1.790	0.073
U_IgG (mg/L)	329.7(3.38,5330)	358.6(3.41,1994)	306.5(4.38,5330)	-2.170	0.353	314.68(4.58,2331.8)	392.72(5,2331.5)	254.23(4.58,1904.1)	-1.470	0.141
24hUTP (g/L)	5.27(0.029,61.68)	4.68(0.386,13.304)	5.74 (0.029,61.68)	-3.991	0.038	4.23(0.02,20.87)	4.43(0.02,16.97)	4.08(0.12,20.87)	-1.090	0.037
RBC (10^12^/L)	3.87±0.89	3.64±0.78	4.07±0.92	-4.297	0.000	3.85±0.95	3.61±0.79	4.04±1.01	-1.412	0.010
HGB (g/L)	110.05±26.72	103.40±22.34	115.40±28.67	-4.158	0.000	110.08±26.9	103.51±24.03	115.13±27.90	-1.789	0.003
HCT (%)	33.58±7.66	31.47±6.50	35.27±8.08	-4.407	0.001	54.59±7.8	31±7.31	65.18±7.69	-1.651	0.001

Note: DKD = Diabetic Kidney Disease; NDKD = Non-Diabetic Kidney Disease.

**Table 1B:** Clinical Baseline Characteristics of Patients in the Training and Validation Sets

Clinical indicator	Validation set (Transplant Group)					Validation set (Non-Transplant + Transplant Group)				
	Total (n=62)	DKD group (n=8)	NDKDgroup (n=54)	Statistic (χ2/F/Z value)	*P* value	Total (n=314)	DKD group (n=118)	NDKD group (n=196)	Statistic (χ2/F/Z value)	*P* value
Male [cases (%)]	53(85.5)	8(100.0)	45(83.3)	9.326a	0.002	228(72.6)	98(83.1)	130(66.3)	5.158^a^	0.003
Age (years)	52(25,72)	54(33,71)	51(25,72)	-1.879	0.040	50(25,77)	51(30,71)	49(25,77)	-1.205	0.317
Height (m)	1.67(1.49,1.82)	1.65(1.6,1.75)	1.68(1.49,1.82)	0.007	0.003	1.65(1.14,1.86)	1.67(1.4,1.86)	1.64(1.41,1.81)	0.010	0.001
Weight (kg)	68(44,90)	64(45,81.5)	69(44,90)	-1.679	0.093	66.3(18.9,146)	67.9(42,114)	65.2(18.9,146)	-0.170	0.793
BMI (kg/m²)	24.18(18.49,25.87)	23.27(20.17,25.73)	24.32(21.97,26.31)	-0.627	0.531	24.1(13.75,32)	24.39(16.40,32)	23.93(18.91,27.97)	-0.699	0.501
Diabetes time(months)	77(1, 360)	72(1, 300)	78(1, 360)	-2.613	0.009	65(1, 360)	96(1, 312)	46(1, 360)	-9.857	0.001
SBP (mmHg)	135±16	140±13	134±16	-1.606	0.118	140±23	145±24	137±22	5.021	0.001
DBP (mmHg)	82±13	83±8	82±11	-3.660	0.001	84±14	84±12	84±15	-1.308	0.099
PP (mmHg)	53(49,103)	56(49,78)	53(28,103)	-0.737	0.461	56(45,88)	61(54,88)	53(44,75)	-5.856	0.000
UA (μmol/L)	391±86	425±79	374±85	-0.113	0.911	413±114	434±104	401±117	0.388	0.665
UREA (mmol/L)	13.33(1.8,31.3)	14.53(7.3,29.8)	13.16(5.26,26.05)	-2.469	0.014	14.24(2.6,19.8)	18.95(6.65,20.68)	11.41(4.90,19.93)	-5.482	0.002
Scr (umol/L)	214(50,766)	281(118,509)	204(50,760)	-2.837	0.005	247(37,450)	313(44,531)	207(69,434)	-5.963	0.000
DR	1(1.6)	1(12.5)	0(0)	-2.579	0.010	75(23.8)	68(57.6)	7(3.6)	-6.023	0.002
eGFR (mL/min/1.73 m^2^)	24.19(8.9,93.6)	34.39(11.04,35.16)	47.14(8.9,93.6)	-2.726	0.006	52.79(3.54,268)	40.48 (4.49,190.69)	60.21(3.54,268)	-5.126	0.000
IgA (g/L)	2.30(0.17,3.61)	2.74(2.06,3.61)	2.24(0.17,3.61)	-2.469	0.014	2.43(0.17,6.3)	2.69(0.69,6.12)	2.63(0.17,6.3)	-1.002	0.319
ALB (g/L)	35.7±6.0	28.5±6.0	36.8±7.7	1.694	0.011	32.5±7.6	32.7±6.6	32.4±5.1	0.882	0.296
TC (mmol/L)	5.45(2.4,8.6)	5.56(3.4,7.6)	5.43(2.4,8.6)	-1.142	0.032	5.97(2.40,8.65)	5.64(3,7.76)	6.17(2.4,8.65)	-2.995	0.003
TG (mmol/L)	2.32(0.65,9.89)	2.63(1.05,4.48)	2.28(0.65,9.89)	-2.880	0.060	2.21(0.56,10.33)	2.04(0.56,6.31)	2.30(0.62,10.33)	-1.658	0.069
LDL-C (mmol/L)	1.18(0.54,2.33)	1.13(0.57,1.51)	1.19(0.54,2.33)	-1.981	0.000	1.20(0.97,1.51)	1.13(0.93,1.29)	1.26(0.99,1.57)	-2.999	0.000
HDL-C (mmol/L)	3.42(1.38,6.43)	3.56(2.02,5.23)	3.40(1.38,6.43)	-1.360	0.018	1.63(0.52,4.72)	2.10(0.52,4.72)	3.85(1.26,4.13)	-3.421	0.031
Fasting Blood Glu (mmol/L)	5.9(2.8,11)	6.1(2.8,11)	5.9(3.1, 10.7)	-0.107	0.268	6.41(2.6,14.3)	7.03(2.8,14.3)	6.04(2.6,10.59)	-7.103	0.003
HbA1c (%)	7.0(5.0,13.1)	7.0(5.45,10.48)	6.4(5.0,13.1)	-0.854	0.338	6.90(4.00,13.20)	7.14 (4.40,13.20)	6.78(4.0,7.0)	-3.021	0.000
U_κ (mg/L)	8.05(3.65,12.9)	8.85(8.03,8.97)	7.94(3.65,12.9)	-0.677	0.461	8.38(1.28,64.64)	8.97(3.15,27.01)	8.02(1.28,64.64)	-3.523	0.000
U_IgG (mg/L)	180(6.93,664)	293(53,664)	163(6.93,373)	-1.485	0.113	288(4.58,2331.1)	386(5,2331.1)	36.00(4.58,1904.1)	-2.964	0.001
24hUTP (g/L)	2.75 (0.192,8.764)	4.88(3.91,6.01)	2.44(0.19,8.76)	-0.868	0.338	3.94(0.02,20.87)	4.46(0.02,16.97)	3.63(0.12,20.87)	-2.456	0.011
RBC (10^12^/L)	4.04±0.80	3.87±0.99	4.06±0.77	-3.200	0.000	3.89±0.92	3.63±0.81	4.04±0.95	-6.124	0.000
HGB (g/L)	116±23.52	107±21.48	118±23.49	-3.225	0.004	111.39±26.4	103.82±23.88	115.95±26.79	-6.726	0.000
HCT (%)	24.19±6.89	34.39±5.67	47.14±6.92	-2.390	0.001	33.68±7.67	31.07±7.22	35.26±7.50	-7.115	0.003

Note: DKD = Diabetic Kidney Disease; NDKD = Non-Diabetic Kidney Disease.

**Table 1C:** Clinical Baseline Characteristics of Patients in the Training and Validation Sets

Clinical indicator	External validation set 1					External validation set 2				
	Total (n=166)	DKD group (n=74)	NDKD group (n=92)	Statistic (χ2/F/Z value)	*P* value	Total (n=200)	DKD group (n=64)	NDKD group (n=136)	Statistic (χ2/F/Z value)	*P* value
Male [cases (%)]	138(83.1)	59(79.7)	79(85.9)	1.103^a^	0.306	126(63.0)	46(71.9)	80(58.8)	3.180^a^	0.085
Age (years)	51(46,57)	51(45,57)	51(46,57)	-0.356	0.722	54(46,60)	52(45,58)	55(49,61)	-1.609	0.108
Height (m)	1.68(1.62,1.72)	1.66(1.60,1.71)	1.68(1.65,1.72)	-1.725	0.085	1.63(1.58,1.69)	1.64(1.59,1.68)	1.63(1.58,1.70)	-0.197	0.844
Weight (kg)	69.5(61.0,77.6)	66.0(60.0,77.1)	72.1(63.9,78.8)	-1.903	0.057	66.8(59.0,74.0)	67.5(60.4,72.9)	66.3(58.8,74.3)	-0.551	0.581
BMI (kg/m²)	25.14(22.76,27.20)	24.65(22.46,27.01)	25.35(22.79,27.53)	-1.345	0.179	24.93(22.78,26.99)	25.24(22.78,27.55)	24.93(22.77,26.78)	-0.621	0.535
Diabetes time(months)	60.0(16.5,120.0)	90.0(48.0,171.0)	36.0(12.0,72.0)	-4.951	0.000	27.5(2.0,96.0)	90.0(24.0,131.5)	12.0(1.0,50.0)	-5.994	0.000
SBP (mmHg)	136±25	142±28	132±21	2.767	0.006	143±22	149±23	140±21	2.564	0.011
DBP (mmHg)	86±12	85±13	87±11	-0.923	0.358	84±14	82±12	85±15	-1.820	0.071
PP (mmHg)	46(35,59)	54(40,67)	46(34,50)	-4.303	0.000	58(47,70)	67(55,80)	54(45,66)	-4.691	0.000
UA (μmol/L)	397±114	406±103	390±122	0.900	0.369	393±115	410±110	386±117	1.368	0.173
UREA (mmol/L)	6.80(5.38,9.16)	7.71(6.40,11.02)	6.18(4.80,8.24)	-4.251	0.000	7.10(5.18,10.50)	9.35(6.65,12.83)	6.60(4.80,8.60)	-4.686	0.000
Scr (umol/L)	119(91,168)	133(96,203)	110(84,135)	-3.012	0.003	99(74,163)	142(90,210)	87(67,138)	-4.294	0.000
DR	65(39.2)	55(74.3)	10(10.9)	69.314^a^	0.000	89(44.5)	56(87.5)	33(24.3)	70.462^a^	0.000
eGFR (mL/min/1.73 m^2^)	45.6(30.4,64.8)	39.5(24.4,59.1)	49.4(39.8,70.8)	-2.854	0.004	56.0(30.4,82.1)	36.5(23.7,61.3)	63.2(38.7,90.2)	-4.150	0.000
IgA (g/L)	2.47(1.86,3.20)	2.42(1.85,2.79)	2.60(1.89,3.55)	-1.575	0.115	2.52(1.83,3.54)	2.74(1.96,3.64)	2.38(1.83,3.50)	-1.237	0.216
ALB (g/L)	39.8±7.5	37.0±7.6	42.0±6.7	-4.534	0.000	31.3±8.6	30.9±7.2	31.6±9.2	-0.583	0.561
TC (mmol/L)	4.91(3.93,5.88)	5.23(3.95,6.01)	4.70(3.89,5.63)	-1.322	0.186	5.92(4.27,7.60)	5.92(4.18,6.93)	5.92(4.29,8.03)	-0.911	0.362
TG (mmol/L)	1.87(1.25,2.77)	1.93(1.30,2.81)	1.85(1.25,2.59)	-0.53	0.596	1.96(1.28,3.02)	1.96(1.21,3.06)	1.94(1.28,2.98)	-0.321	0.748
LDL-C (mmol/L)	2.82(2.11,3.66)	3.03(2.05,3.87)	2.79(2.16,3.56)	-0.967	0.334	3.54(2.58,4.65)	3.49(2.60,4.41)	3.57(2.53,5.00)	-0.630	0.529
HDL-C (mmol/L)	1.01(0.86,1.22)	1.04(0.88,1.23)	1.00(0.84,1.22)	-0.616	0.538	1.19(0.99,1.52)	1.11(0.95,1.29)	1.25(1.02,1.61)	-2.624	0.009
Fasting Blood Glu (mmol/L)	6.93(5.59,9.12)	8.36(5.81,10.52)	6.36(5.49,7.40)	-3.908	0.000	6.11(5.21,7.80)	7.18(5.52,9.76)	5.90(5.08,7.04)	-3.456	0.001
HbA1c (%)	7.20(6.43,8.70)	7.60(6.58,9.73)	6.80(6.18,7.53)	-3.77	0.000	6.80(6.10,7.60)	7.20(6.30,8.50)	6.65(6.08,7.30)	-2.512	0.012
U_κ (mg/L)	37.90(16.90,101.81)	73.50(33.58,137.00)	23.93(10.80,58.52)	-4.902	0.000	61.60(32.20,127.25)	97.30(47.30,138.50)	48.50(27.55,101.254)	-2.886	0.004
U_IgG (mg/L)	56.1(9.8,58.6)	57.2(32.3,58.6)	17.3(5.4,57.5)	-4.035	0.000	121.0(50.2,320.0)	216.0(92.2,418.0)	100.2(43.4,220.8)	-3.504	0.000
24hUTP (g/L)	0.53(0.10,2.23)	1.41(0.30,2.88)	0.19(0.08,1.23)	-4.192	0.000	0.55(0.25,1.13)	0.75(0.31,1.58)	0.49(0.22,1.11)	-2.022	0.043
RBC (10^12^/L)	4.41±0.79	4.11±0.75	4.64±0.75	-4.560	0.000	4.20±0.85	3.88±0.80	4.34±0.83	-3.691	0.000
HGB (g/L)	128.99±23.99	119.74±23.79	136.42±21.54	-4.733	0.000	120.03±23.38	110.97±23.07	124.29±22.36	-3.890	0.000
HCT (%)	38.52±6.79	35.72±6.69	40.78±6.01	-5.118	0.000	36.06±7.14	33.24±6.58	37.39±7.04	-3.966	0.000

Note: DKD = Diabetic Kidney Disease; NDKD = Non-Diabetic Kidney Disease.

**Table 2:** Results of Univariate and Multivariate Logistic Regression Analysis on the Training Set

Variable	Univariate Analysis		Multivariate Analysis	
	OR (95%CI)	*P* value	OR (95%CI)	*P* value
Sex (Male/Female)	2.887 (1.693, 5.047)	0.001	12.577(1.144,169.707)	0.043
Age (Years)	1.021 (1.000, 1.042)	0.049	1.021(1.000,1.119)	0.038
Height (m)	0.005 (0.000, 0.119)	0.001		
Diabetes duration (Months)	0.985 (0.981, 0.989)	0.001	0.988(0.976,0.999)	0.046
SBP (mmHg)	0.988 (0.977, 0.998)	0.019		
PP (mmHg)	0.971 (0.957, 0.985)	0.001		
UA (umol/L)	0.997 (0.994, 0.999)	0.002	2.232(1.096,5.241)	0.040
UREA (mmol/L)	0.927 (0.897, 0.956)	0.001		
Scr (umol/L)	0.997 (0.996, 0.999)	0.001		
DR	13.169 (7.663, 25.188)	0.001	0.014(0.000,1.000)	0.076
eGFR (mL/min/1.73 m^2^)	1.017 (1.004, 1.032)	0.013		
ALB (g/L)	0.959 (0.928, 0.990)	0.010		
U_κ (mg/L)	0.846 (0.777, 0.916)	0.001		
TC (mmol/L)	1.143 (1.049, 1.258)	0.004		
TG (mmol/L)	1.083 (0.972, 1.228)	0.177		
HDL-C (mmol/L)	2.008 (1.232, 3.433)	0.008		
LDL-C (mmol/L)	1.251 (1.092, 1.454)	0.002	0.484(0.275,0.749)	0.003
Fasting Blood Glu (mmol/L)	0.918 (0.843, 0.996)	0.041		
HbA1c (%)	0.846 (0.721, 0.985)	0.034		
Urine-IgG (mg/L)	1.000(0.999, 1.000)	0.353		
24hUTP (mg/L)	1.062 (1.008, 1.131)	0.038		
RBC (10^12^/L)	1.778 (1.349, 2.375)	0.001		
HGB (g/L)	1.018 (1.009, 1.027)	0.001		
HCT (%)	940.134 (37.886, 27122.225)	0.001		

**Table 3:** Performance of the Predictive Model in the Training and Verification Sets

Indicator	Training set	Validation set (Non-Transplant Group)	Validation set (Transplant Group	Validation set (Non-Transplant + Transplant Group)	External validation set 1	External validation set 2
Sample size	290	252	62	314	166	200
Discrimination						
C-statistic (AUC 95%CI)	0.906(0.8643-0.9478)	0.881(0.8379-0.9242)	0.516(0.2934-0.739)	0.840(0.7944-0.8862)	0.836(0.7704-0.9008)	0.871(0.8190-0.9221)
Sensitivity (%)	80.5	76.1	57.4	75.0	85.9	78.7
Specificity (%)	92.4	88.2	62.5	80.5	73.0	86.9
PPV	93.4	89.3	91.2	86.6	79.8	93.0
NPV	80.0	74.0	17.9	66.0	80.6	64.6
Calibration Hosmer-Lemeshow test P value	0.287	0.126	7.81E^-11^	0.003	0.895	0.268
Brier score	0.104	0.142	0.285	0.170	0.176	0.153

## Abbreviations

DKD: diabetic kidney disease
NDKD: non-diabetic kidney disease
T2DM: type 2 diabetes mellitus
DM: diabetes mellitus
MIX: mixed type
BMI: body mass index
DR: diabetic retinopathy
Scr: serum creatinine
UREA: blood urea
UA: uric acid
eGFR: estimated glomerular filtration rate
ALB: albumin
IgA: immunoglobulin A
TC: total cholesterol
TG: triglyceride
HDL-C: high density lipoprotein cholesterol
LDL-C: low density lipoprotein cholesterol
Glu: glucose
HbA1c: glycated hemoglobin
U_κ: urinary κ-chain
U_IgG: urinary immunoglobulin G
24hUTP: 24-hour urinary total protein amount
RBC: red blood cell count
HGB: hemoglobin
HCT: hematocrit
ORs: odds ratios
PPV: positive likelihood ratio
NPV: negative likelihood value
