# Supplementary material for: Study on Predictive Models for Differential Diagnosis of Diabetic Kidney Disease and Non-Diabetic Kidney Disease Based on Clinical and Biochemical Indicators
Source: Int J Med Sci. 2025 Sep 27;22(15):4119–30. doi: 10.7150/ijms.115709 (PMC12492356; doi:10.7150/ijms.115709)
Supplement: Supplementary file 1 — Supplementary tables. [file ijmsv22p4119s1.pdf]

Supplementary Table 1. Distribution of Pathological Types in NDKD and MIX Patients in the Training and Validation Sets [cases (%)]

Pathological Type	Training set			Validation Set (Non-Transplant Group)			Validation Set (Transplant Group)			Validation Set (Non-Transplant + Transplant Group)			External validation set 1			External validation set 2		
	MIX（n=40）	NDKD（n=161）	ALL（n=330）	MIX（n=35）	NDKD（n=142）	ALL（n=287）	MIX（n=11）	NDKD（n=54）	ALL（n=73）	MIX（n=46）	NDKD（n=96）	ALL（n=360）	MIX（n=35）	NDKD（n=92）	ALL（n=201）	MIX（n=0）	NDKD（n=136）	ALL（n=200）
Diabetic Nephropathy			119（36.1）			98(34.1)			5（6.8）			103（28.6）			49 (24.4)			56 (28.0)
Diabetic Nephropathy with Ischemic or Tubulointerstitial Damage			10（3.0）			12(4.2)			3（4.1）			15（4.2）			25 (12.4)			8 (4.0)
Membranous Nephropathy	13（32.5）	56（34.8）	69（20.9）	6（17.1）	45（31.7）	51(17.8)				6（13.0）	45（23.0）	51（14.2）	2 (5.7)	11 (12.0)	13 (6.5)		57 (41.9)	57 (28.5)
IgA Nephropathy	5（12.5）	29（18.0）	34（10.3）	5（14.3）	31（21.8）	36(12.5)	3（27.3）	10（18.5）	13（17.8）	8（17.4）	41（20.9）	49（13.6）	6 (17.1)	15 (16.3)	21 (10.4)		28 (20.6)	28 (14.0)
Hypertensive Nephropathy	14（35.0）	14（8.7）	28（8.5）	7（20.0）	7（4.9）	14(4.9)	2（18.2）	1（1.9）	3（4.1）	9（19.6）	8（4.1）	17（4.7）	12 (34.3)	27 (29.3)	39 (19.4)		3 (2.2)	3 (1.5)
Minimal Change Disease		8（5.0）	8(2.4）	3（8.6）	3（2.1）	6(2.1)	1（9.1）		1（1.4）	4（8.7）	3（1.5）	7（1.9）	2 (5.7)	23 (25.0)	25 (12.4)		8 (5.9)	8 (4.0)
Focal Segmental	1	11	12	3	11（7.7）	14(4.9)	1	3	4	4	14（7.1）	18（5.0）					13	13

Glomerulosclerosis	（2.5）	（6.8）	（3.6）	（8.6）			（9.1）	（3.33）	（5.5）	（8.7）							(9.6)	(6.5)
Sclerosing Glomerulonephritis	1（2.5）	2（1.2）	2（0.9）		1（0.7）	1(0.3)		2（3.7）	2（2.7）		3（1.5）	3（0.8）	2 (5.7)	5 (5.4)	7 (3.5)		4 (2.9)	4 (2.0)
Mesangial Proliferative Glomerulonephritis		5（3.1）	5（1.5）	2（5.7）	2（1.4）	4(1.4)		1（1.9）	1（1.4）	2（4.3）	3（1.5）	5（1.4）	1 (2.9)	3 (3.3)	4 (2.0)		6 (4.4)	6 (3.0)
Acute and Chronic Tubulointerstitial Injury	1（2.5）	13（8.1）	14（4.2）	6（17.1）	4（2.8）	10(3.5)	1（9.1）	5（9.3）	6（8.2）	7（15.2）	9（4.6）	16（4.4）	9 (25.7)	2 (2.2)	11 (5.5)		5 (3.7)	5 (2.5)
Immune Complex-Mediated Glomerulonephritis		2（1.2）	2（0.6）	1（2.9）	1（0.7）	2(0.7)	3（27.3）	2（3.7）	5（6.8）	4（8.7）	3（1.5）	7（1.9）					1 (0.7)	1 (0.5)
Vasculitis	1（2.5）	4（2.5）	5（1.5）					2（3.7）	2（2.7）		2（1.21）	2（0.6）					3 (2.2)	3 (1.5)
Amyloidosis	1（2.5）	3（1.9）	4（1.2）		2（1.4）	2(0.7)					2（1.21）	2（0.6）					2 (1.5)	2 (1.0)
Light Chain-Related Kidney Damage		3（1.9）	3（0.9）		3（2.1）	3(1.0)					3（1.5）	3（0.8）						
Transplant Kidney Rejection	3（7.5）	3（1.9）	6（1.8）		3（2.1）	3(1.0)		25（46.3）	25（34.2）		28（14.3）	28（7.8）						
Lupus Nephritis		2（1.2）	2（0.6）		11（7.7）	11(3.8)		1（1.9）	1（1.4）		12（6.1）	12（3.3）					5 (3.7)	5 (2.5)

Crescentic Glomerulonephritis	2（1.2）	2(0.6）	1（2.9）	12（8.5）	13(4.5)			1（2.2）	12（6.1）	13（3.6）					
Henoch-Schönlein Purpura Nephritis	1（0.6）	1(0.3）	1（2.9）	3（2.1）	4(1.4)			1（2.2）	3（1.5）	4（1.1）		1 (1.1)	1 (0.5)		
Thrombotic Microangiopathy	1（0.6）	1(0.3）		2（1.4）	2(0.7)				3（1.5）	2（0.6）					
Other	2（1.2）	2(0.6）		1（0.7）	1(0.3)	2（3.7）	2（2.7）		3（1.5）	3（0.8）	1 (2.9)	5 (5.4)	6 (3.0)	1 (0.7)	1 (0.5)

Note: NDKD = Non-Diabetic Kidney Disease; MIX = Diabetic Kidney Disease combined with Non-Diabetic Kidney Disease.

Supplementary Table 2. Assessment of multicollinearity among HbA1c, Fasting Glucose, Diabetes Duration.

Model		Non standardized coefficient		Standardization coefficient	t	Significance	Collinearity statistics	
		B	Standard error	Beta			Tolerance	VIF
1	(Constant)	0.963	0.128		7.502	0.000		
	Diabetes duration	-0.003	0.000	-0.431	-8.053	0.000	0.971	1.030
	Glu	-0.009	0.009	-0.052	-0.961	0.338	0.938	1.066
	HbA1c	-0.018	0.018	-0.056	-1.032	0.303	0.937	1.068

Supplementary Table 3. Assessment of multicollinearity among UA, Age, LDL, Sex, DR, Diabetes Duration.

Model		Non standardized coefficient		Standardization coefficient	t	Significance	Collinearity statistics	
		B	Standard error	Beta			Tolerance	VIF
1	(Constant)	0.274	0.149		1.836	0.067		
	sex	0.154	0.049	0.143	3.116	0.002	0.959	1.043
	age	0.003	0.002	0.075	1.606	0.109	0.932	1.073
	diabetes duration	-0.002	0.000	-0.286	-5.883	0.000	0.848	1.179
	diabetic_retinopathy	0.437	0.050	0.422	8.656	0.000	0.844	1.185
	UA	0.000	0.000	-0.081	-1.762	0.079	0.945	1.058
	LDL	0.019	0.011	0.078	1.699	0.090	0.943	1.060

Supplementary Material-Stepwise

R language：

```
library(readr)
data <- read_csv("E:/R-data2/data7-7stepwise.csv")
data<-na.omit(data)

model.old<-glm(group~., data=data,family=binomial())

library(MASS)

model.both<-stepAIC(model.old,direction ="both")
```

```
Start:  AIC=223.78
group ~ sex + age + BMI + diabetes_duration + diabetic_retinopathy + 
    UA + LDL

                       Df Deviance    AIC
- BMI                   1   208.20 222.20
- UA                    1   209.46 223.46
<none>                      207.78 223.78
- LDL                   1   209.81 223.81
- age                   1   210.35 224.35
- sex                   1   221.11 235.11
- diabetes_duration     1   233.05 247.05
- diabetic_retinopathy  1   257.88 271.88

Step:  AIC=222.2
group ~ sex + age + diabetes_duration + diabetic_retinopathy + 
    UA + LDL

                       Df Deviance    AIC
- UA                    1   209.67 221.67
<none>                      208.20 222.20
- LDL                   1   210.29 222.29
- age                   1   210.71 222.71
+ BMI                   1   207.78 223.78
- sex                   1   221.34 233.34
- diabetes_duration     1   233.07 245.07
- diabetic_retinopathy  1   261.21 273.21

Step:  AIC=221.67
group ~ sex + age + diabetes_duration + diabetic_retinopathy + 
    LDL
```

```
                    Df Deviance    AIC
<none>                   209.67 221.67
+ UA                 1   208.20 222.20
- age                1   212.25 222.25
- LDL                1   212.27 222.27
+ BMI                1   209.46 223.46
- sex                1   224.20 234.20
- diabetes_duration    1   235.15 245.15
- diabetic_retinopathy  1   264.67 274.67
```
